# Circulating MicroRNA-15a Associates With Retinal Damage in Patients With Early Stage Type 2 Diabetes

**DOI:** 10.3389/fendo.2020.00254

**Published:** 2020-04-23

**Authors:** Elena Sangalli, Elena Tagliabue, Lucia La Sala, Francesco Prattichizzo, AnnaChiara Uccellatore, Daniela Spada, Fabrizio Lorino, Paola de Candia, Silvia Lupini, Laura Cantone, Chiara Favero, Paolo Madeddu, Valentina Bollati, Stefano Genovese, Gaia Spinetti

**Affiliations:** ^1^IRCCS MultiMedica, Milan, Italy; ^2^Department of Biomedical and Clinical Science L. Sacco, University of Milan, Milan, Italy; ^3^EPIGET Lab, Department of Clinical Sciences and Community Health, University of Milan, Milan, Italy; ^4^Department of Translational Health Sciences, Bristol Medical School, University of Bristol, Bristol, United Kingdom; ^5^Centro Cardiologico Monzino IRCCS, Milan, Italy

**Keywords:** prediction and prevention of type 2 diabetes, retinopathy, microvascular disease, microRNA-15a, extracellular vesicles

## Abstract

Circulating microRNAs are potential biomarkers of type 2 diabetes mellitus (T2DM) and related complications. Here, we investigated the association of microRNA-15a with early retinal damage in T2DM. A cohort of untreated subjects screened for intermediate/high risk of T2DM, according to a score assessment questionnaire, and then recognized to have a normal (NGT) or impaired (IGT) glucose tolerance or T2DM was studied. The thickness of the ganglion cell complex (GCC), an early marker of retinal degeneration anteceding overt retinopathy was assessed by Optical Coherence Tomography. Total and extracellular vesicles (EV)-associated microRNA-15a quantity was measured in plasma by real time PCR. MicroRNA-15a level was significantly higher in subjects with IGT and T2DM compared with NGT. MicroRNA-15a abundance was correlated to body mass index and classical diabetes biomarkers, including fasting glucose, HbA1c, insulinemia, and HOMA-IR. Moreover, GCC thickness was significantly reduced in IGT and T2DM subjects compared with NGT controls. Importantly, total microRNA-15a correlated with GCC in IGT subjects, while in T2DM subjects, EV-microRNA-15a negatively correlated with GCC, suggesting that microRNA-15a may monitor initial retinal damage. The assessment of plasma microRNA-15a may help refining risk assessment and secondary prevention in patients with preclinical T2DM.

## Introduction

The number of adults affected by type 2 diabetes mellitus (T2DM) and the proportion of T2DM patients with vascular and neurological complications is expected to grow substantially in future decades (1). Therefore, prognostic biomarkers capable of establishing clinical practice recommendations for patients who have impaired glucose tolerance (IGT) and are at increased risk of developing T2DM-releated complications are urgently needed (2). High levels of plasma glucose are associated with increased insulin production by the pancreatic beta cells and insulin resistance in peripheral tissues, resulting in endothelial dysfunction, microvascular damage, and neural degeneration. In the context of retinopathy, the neurodegenerative theory hypothesizes early anatomical changes occur in the ganglionic cell complex (GCC), prior to the onset of microangiopathy. GCC can be assessed by the specialist using Optical Coherence Tomography (OCT), a test that uses light waves to take cross-sectional pictures of the retina. On the other hand, there is a need to identify reliable biomarkers from an easily accessible source that could generate cost-effective assays feasible for routine screening. Blood-based biomarkers may offer a non-invasive strategy to improve risk assessment in individuals with preclinical or uncomplicated T2DM (3). We and others reported that specific circulating microRNA signatures may help predicting or detecting the development and progression of T2DM at an early stage (4, 5). MicroRNAs are small (20–22 bp) non-coding RNA molecules that act as post-transcriptional regulators of gene expression in the cell of origin as well as in neighbor and distant cells. After secretion and transport through the circulation, microRNAs are shuttled to target cells by proteins (e.g., Ago-2 and high density lipoproteins) and extracellular vesicles (EVs) (6, 7). High levels of microRNA-15a associate with an increased risk of post-revascularization amputation in T2DM subjects with severe peripheral vascular complications (8). Moreover, a recent study demonstrated that high levels of extracellular vesicles (EV)-associated microRNA-15a can be found in plasma of subjects with diabetic retinopathy (9). Here, we report a pilot observational study aimed to provide initial evidence for the usefulness of total and EV-microRNA-15a as an early biomarker of complications in patients with preclinical T2DM.

## Results

### Clinical and Laboratory Characteristics

**Figure 1:**
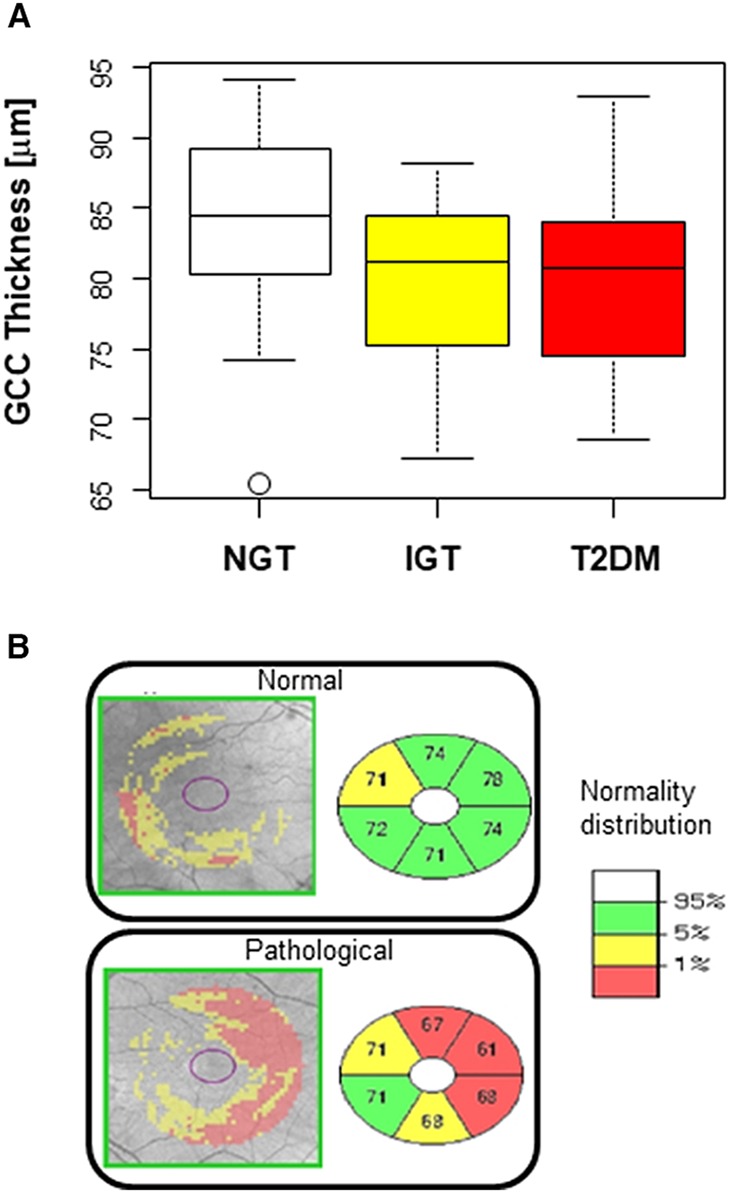
Thickness of the ganglionic cell complex (GCC) decrease in subject with IGT and T2D. **(A)** Box plot of GCC thickness (μm) in the three diagnostic groups. Overall *p* = 0.02. T2DM vs. NGT *p* = 0.048. IGT vs. NGT *p* = 0.04. Boxes are bordered at the 25th and the 75th percentile of the predictor variable and a median line at the 50th percentile. Whiskers extend from the box to the upper and lower adjacent values and are capped with an adjacent line. Points below and above the whiskers (10–90th percentile) are drawn as individual dots. **(B)** Representative images and color grade (white indicating normal and red most reduced GCC thickness).

Characteristics of the 76 enrolled subjects are shown in (Table 1). Subjects in the IGT and T2DM groups tended to be older compared with control therefore age was always taken into consideration for subsequent analyses. The analysis of GCC was performed in a subgroup of patients who gave consent or were eligible for an OCT test, according to the criteria described in the Methods section. In total, we analyzed 33 eyes of NGT subjects, 31 of IGT, and 30 of T2DM. Results indicate the average GCC thickness was significantly decreased in subjects with IGT or T2DM as compared with NGT (Figures 1A,B).

**Table 1:** Anamnestic and laboratory tests data characterizing the study population.

	**NGT (*N* = 26)**		**IGT (*N* = 24)**		**T2DM (*N* = 26)**		
	***n (%)***		***n (%)***		***n (%)***		p^**^
	***mean***	***SD***	***mean***	***SD***	***mean***	***SD***	
SEX (males)	9 (34.6)		12 (50.0)		10 (38.5)		0.5°
Age (years)	56	9.6	62.5	8.3	61.2	7.8	0.02^*^
FPG (mg/dL)	82.1	8	94.5	10	110.2	14.7	**<0.0001**
OGTT (mg/dL)	99.8	22.9	157.8	16.1	238.3	39.9	**<0.0001**
HbA1c (%)	5.6	0.3	6.2	0.4	6.6	0.6	<0.0001^*^
HbA1c (mmol/mol)	38.1	2.8	43.7	4	48.9	6.4	**0.01**
Insulin (mlU/L)	16.6	22.4	19.6	30.5	20.5	20.4	**0.0005**
HOMA-IR	3.4	4.6	4.7	7.5	5.7	6	**0.02**
SBP (mmHg)	128.5	12.3	132.7	20.2	132.5	15.1	0.4^*^
DBP (mmHg)	73.1	11.8	79	11	80.1	13	0.5
Heart rate (bpm)	69.8	8.8	68.8	7.8	73	7.2	**0.01**
BMI (Kg/m^2^)	25.2	3.7	26.6	3.6	29	5.7	0.09
Waist circumference (cm)	92.4	11.8	98.3	8.5	100.1	12.2	0.5
Total cholesterol (mg/dL)	201.5	32.8	215.2	34.5	202.3	31.6	0.2
HDL cholesterol (mg/dL)	57.3	12.4	53.6	13.5	52.1	13.1	0.3^*^
LDL cholesterol (mg/dL)	122.3	28.4	136.2	33.1	124.6	27.1	0.3^*^
Triglycerides (mg/dL)	109.6	63.5	127.2	54.8	128.3	63.5	0.2^*^
Microalbuminuria (mg/dL)	6.5	5.2	29.2	83	18.3	32.1	0.2

Oral glucose tolerance test (OGTT) refers to plasma glucose level measured 2 h post 75 gr of glucose load. FPG, fasting plasma glucose; BMI, body mass index; SBP, sistolic blood pressure; DBP, diastolic blood pressure.The reported p-values refer to ° chi-squared test

* F test

** *Kruskal-Wallis test. Significant p-values are in bold*.

### High Abundance of Circulating microRNA-15a in Subjects With Newly-Diagnosed T2DM

**Figure 2:**
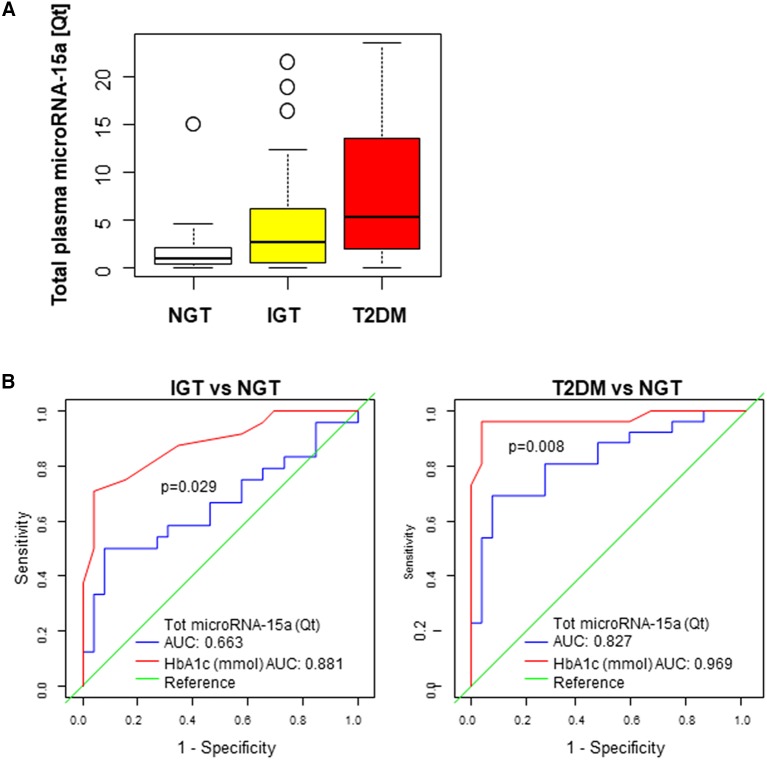
IGT and T2DM subjects bear high levels of circulating microRNA-15a. **(A)** Box plot of plasma microRNA-15a quantity in the three diagnostic groups. Overall *p* = 0.001. T2DM vs. NGT *p* < 0.0001. IGT vs. NGT *p* = 0.01. Boxes are bordered at the 25th and the 75th percentile of the predictor variable and a median line at the 50th percentile. Whiskers extend from the box to the upper and lower adjacent values and are capped with an adjacent line. Points below and above the whiskers (10–90th percentile) are drawn as individual dots. **(B)** Diagnostic accuracy of microRNA-15a. ROCcurves for plasma (tot) microRNA-15a quantity (Qt) and HbA1c (mmol) in predicting T2DM and IGT. AUC and *p*-values for comparison between curves are reported.

By quantitative RT-PCR, we measured the levels of microRNA-15a (normalized by an exogenous spike-in RNA, Cel-microRNA-39) in the plasma of subjects with newly-diagnosed T2DM (*n* = 26), IGT (*n* = 24), or NGT (*n* = 26). Circulating levels of total plasma microRNA-15a were significantly different in the three groups (overall *p* = 0.001), after adjustment by age and sex. Moreover, a significant difference was observed in the comparison of T2DM with NGT (*p* < 0.0001) (Figure 2A). In addition, we found a significant association between the microRNA-15a and markers of altered glucose metabolism, including HbA1c, plasma glucose, insulin, and HOMA-IR (Table 2, left).

**Table 2:** Circulating microRNA-15a correlation with characteristics of the study population.

	**Total plasma microRNA-15a**			**EV microRNA-15a**		
	***N***	rho^*^	***p***	***N***	rho^*^	***p***
Age (years)	76	0.04	0.7	55	0.04	0.7
FPG (mg/dL)	76	0.3	**0.01**	55	0.3	**0.03**
OGTT (mg/dL)	76	0.4	**<0.0001**	55	0.4	**0.002**
HbA1c (%)	76	0.3^**^	**0.02**	55	0.4	**0.01**
HbA1c (mmol/mol)	76	0.4	**0.002**	55	0.4	**0.01**
Insulin (mlU/L)	73	0.3	**0.01**	55	0.4	**0.002**
HOMA-IR	73	0.3	**0.003**	55	0.4	**0.002**
SBP (mmHg)	76	0.3	**0.01**	55	0.3	0.05
DBP (mmHg)	76	0.3	**0.002**	55	0.3	**0.048**
Heart rate (bpm)	76	0.1	0.3	55	0.03	0.8
BMI (Kg/m^2^)	76	0.2^**^	**0.045**	55	0.3	**0.04**
Waist circumference (cm)	76	0.2^**^	**0.046**	55	0.3	0.06
Total cholesterol (mg/dL)	76	−0.01^**^	0.9	55	0.04	0.8
HDL cholesterol (mg/dL)	76	−0.2	0.1	55	−0.1	0.4
LDL cholesterol (mg/dL)	76	−0.01^**^	0.9	55	0.04	0.8
Triglycerides (mg/dL)	76	0.2	0.1	55	0.2	0.1
Microalbuminuria (mg/dL)	74	0.1	0.4	55	−0.07	0.6

*Oral glucose tolerance test (OGTT) refers to plasma glucose level measured 2 h post 75 gr of glucose load. FPG, fasting plasma glucose. DBP, diastolic blood pressure. SBP, sistolic blood pressure*.

The reported p-values refer to

* Spearman's correlation with miR expression

** *Pearson's correlation coefficient with miR expression. Significant p-values are in bold*.

To determine the value of microRNA-15a as a biomarker for diagnosis of IGT orT2DM, the receiving operator characteristic (ROC) curves were drawn and the area under the curve (AUC) calculated (Figure 2B). Results demonstrate the diagnostic accuracy of microRNA-15a for T2DM (AUC = 0.83, 95% CI 0.71–0.94) and IGT (AUC = 0.66, 95% CI 0.51–0.82). In both cases, microRNA-15a has a limitative discriminative power with respect to HbA1c (*p*-value for comparison between ROC curves 0.008 and 0.029, respectively).

### The Abundance of EV-microRNA-15a Negatively Correlates With GCC Thickness in T2D Subjects

**Figure 3:**
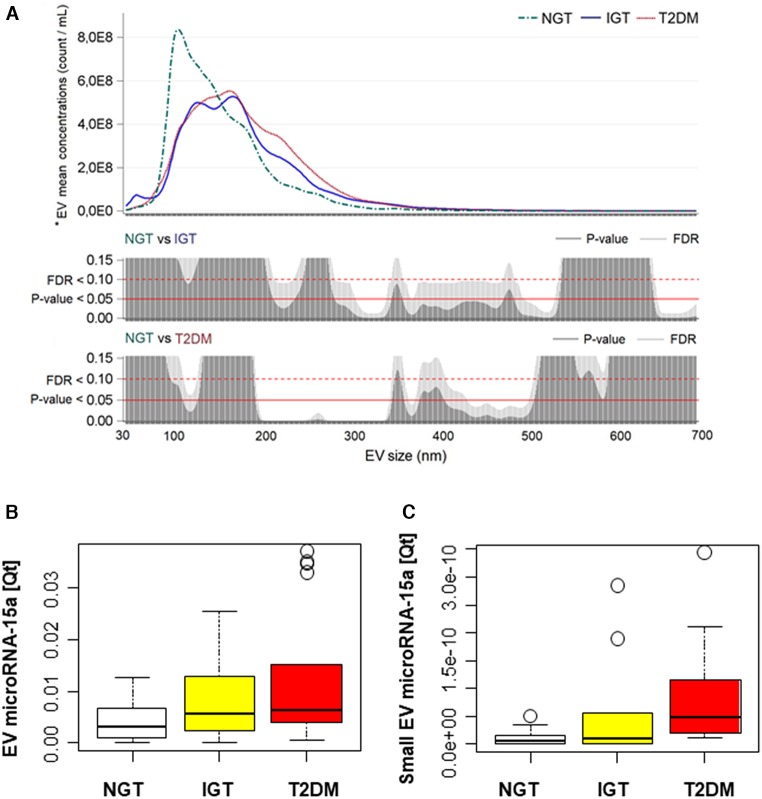
T2DM associates with changes in extracellular vesicles (EVs) size distribution and microRNA-15a content. **(A)** EVs have been studied in a subgroup of subjects: NGT, *N* = 9; IGT, *N* = 10; T2DM, *N* = 10. ^*^ Reported geometric means were adjusted for age and sex. Plots showing for each diagnostic group (NGT, IGT, and T2DM) the distribution of mean vesicle concentrations for each size; vertical bar charts represent FDR and *P*-value for each size comparison; the red line indicates *P* = 0.05; the red dot line indicates FDR = 0.10. **(B,C)** Box plot of EV- and small EV–microRNA-15a quantity in the three diagnostic groups. EV: NGT, *N* = 20; IGT, *N* = 17; T2DM, *N* = 18. Small EV microRNA was obtained by normalizing the quantity of miRNA by the small EV concentration: NGT, *N* = 9; IGT, *N* = 10; T2DM, *N* = 10. Boxes are bordered at the 25th and the 75th percentile of the predictor variable and a median line at the 50th percentile. Whiskers extend from the box to the upper and lower adjacent values and are capped with an adjacent line. Points below and above the whiskers (10–90th percentile) are drawn as individual dots.

To explore if EVs-contained microRNA-15a has a different diagnostic value compared to its whole circulating plasma level, we isolated circulating EVs with size-exclusion chromatography. We found that NGT subjects were characterized by a higher concentration of smaller vesicles, with a peak at 93 nm. When comparing IGT and T2DM subjects, the size distribution was similar, but the small size EV component was present only in the IGT group (Figure 3A). Next, we assessed the level of microRNA-15a contained in EVs. Results indicate the quantity of EV-microRNA-15a is different between the three diagnostic groups (overall *p* < 0.05). Particularly, T2DM subjects presented significantly higher values if compared with NGT subjects (*p* = 0.003) (Figure 3B). Of note, T2DM subjects also showed a significant increase in EV-miRNA-15a quantity embedded in small size EV (30–100 nM in size) (*p* = 0.02 vs. NGT) (Figure 3C). Moreover, similarly to what we observed for total circulating microRNA-15a, EV-microRNA-15a levels associate with clinical parameter of altered glucose metabolism (Table 2, right).

**Figure 4:**
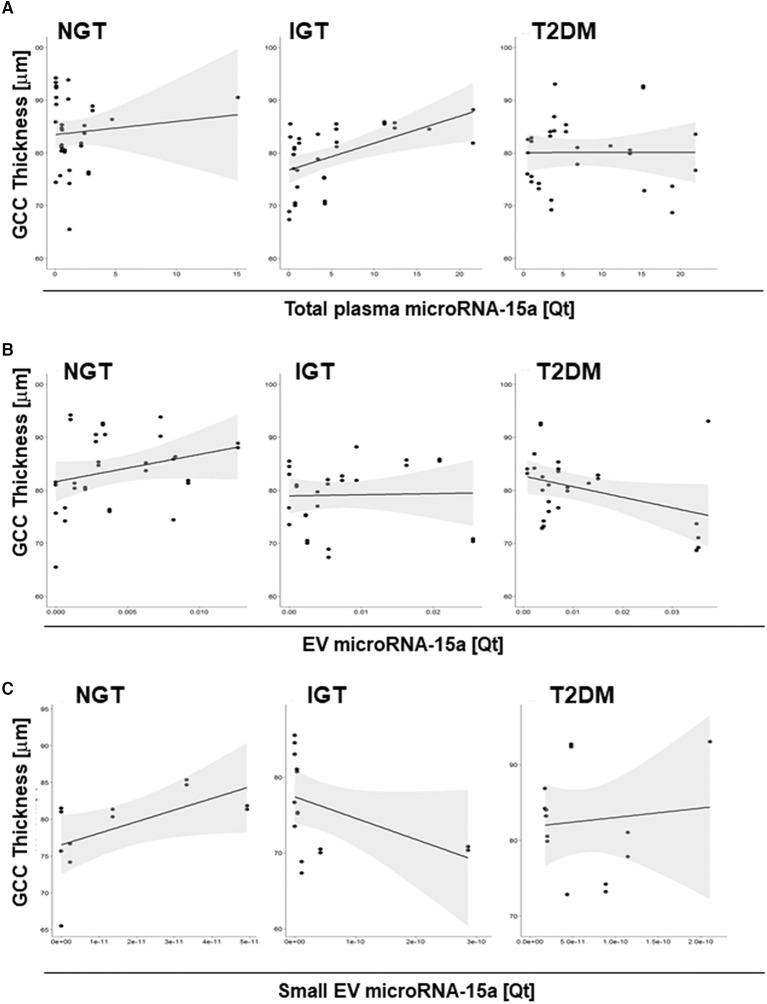
Correlation between circulating microRNA-15a and retinal damage. Scatter plots for the correlation between **(A)** total plasma microRNA-15a quantity (Qt), **(B)** EV-microRNA-15a quantity (Qt), **(C)** small EV-microRNA-15a quantity (Qt) and CGG thickness (μm) in the three diagnostic groups.

Finally, we analyzed the correlation between total plasma-microRNA-15a or EV-microRNA-15a and GCC (Figure 4). Adjusting by age and sex no association was found between total plasma microRNA-15a and GCC in T2DM, but a significant correlation was observed in IGT (*p* = 0.0094 *rho* = 0.474). Moreover, results indicate a significant positive correlation of EV-microRNA-15 quantity and GCC thickness in NGT (*p* = < 0.0001, *rho* = 0.677), but not in T2D (*p* = 0.062, *rho* = −0.37) and IGT (*p* = 0.87, *rho* = 0.034) subjects. Similarly, the fraction of small EV-microRNA quantity positively correlate with GCC in NGT (*p* = 0.0001, *rho* = 0.95), but shows significant inverse correlation in IGT (*p* = 0.0002, *rho* = −0.82).

## Discussion

Here, we showed that circulating total plasma and EV-microRNA-15a increases in subjects with T2DM and associates with clinical parameters of altered glucose metabolism. Differences with a previous report that showed decreased circulating microRNA-15a in diabetes may be due at least in part to the different internal reference used (10). Importantly, in our study in IGT, small EV-microRNA-15a quantity associates with neuronal damage in the retina (decreased thickness of the GCC), an alteration associated with early stage of retinopathy and could represent useful biomarkers at the early phase of the diabetic retinopathy. Interestingly, this association was not observed when using whole circulating levels of microRNA-15a, supporting the notion that small EV-contained miRNAs provide different information when compared to plasmatic microRNAs (11). Data showed for the first time that GCC is decreased already in subjects with IGT and patients with previously undiagnosed T2DM. This is in line with the emerging neuropathic theory of diabetic retinopathy that hypothesizes an earlier neuronal damage anticipating the development of the glucose-mediated vascular injury eventually leading to retinal dysfunction and vision loss (12). Although the organ/cellular source of circulating microRNAs is difficult to decipher, initial evidence suggests microRNA-15a is produced within pancreatic beta cells, with its transcription being increased in overt T2DM (13). As recently shown, EV-associated microRNA-15a could be delivered to the retina triggering reactive oxygen species production and activation of the pro-apoptotic pathways (9). On the other side, another report showed that microRNA-15a is downregulated within the diabetic retina, with subsequent activation of pro-inflammatory and pro-angiogenic pathways (14). Similarly, an *in vitro* study showed that high glucose conditions decreased the expression of microRNA-15a in cultured human retinal endothelial cells, promoting the pro-inflammatory signaling of IL-1β, TNFα, and NF-κB (15). These discrepancies highlight a potential tissue-dependent regulation of microRNA-15a in the diabetic environment. On the other side, they support a key role for this microRNA in the pathogenesis of diabetic retinopathy, an observation that might eventually reinforce its potential as a functional marker of the disease.

MicroRNAs and their shuttles are currently being explored for their ability to provide information about complex pathological traits or multi-factorial diseases, such as T2DM complications (16). Indeed, given their capacity to be finely modulated by a wide variety of T2DM-relvant triggers, they represent an ideal interface between environmental stimuli and the genetic background, possibly providing additional information compared to conventional risk factors (17). On the other side, EVs isolation and miRNA dosages are not commonly used in routine clinical practice, mainly to the long time required for analysis and the lack of internationally accepted method for standardization. These and other aspects must be implemented before miRNAs can represent an additional tool for diagnostic purposes.

Circulating microparticles concentration reportedly increases in subjects with diabetes and carry a specific signature of microRNAs (18). We here demonstrated that diabetic EVs are enriched in larger particles that could reflect an increase in cellular damage in diabetes (19). Interestingly, we detected a peak of small, exosome-like, vesicles only in IGT donors potentially associated with inflammation (20).

Our study limit resides in its observational nature and relatively small sample sizewhich does not allow definitive conclusion and proper adjustments for multiple risk factors. On the other hand, it identifies a previously unknown association between microRNA-15a and early retinal alterations in preclinical diabetes. This observation can have a significant relevance for the refinement of risk stratification and secondary prevention of T2DM-associated complications.

## Materials and Methods

### Study Protocol

NGT, IGT and T2DM subjects were consecutively selected based on plasma glucose (PG) levels after 2 h of a 75 gr glucose load (OGTT) among those at intermediate/high risk for T2DM, according to a risk score assessment questionnaire (21) enrolled in a clinical observational study conducted at IRCCS MultiMedica, Milan, Italy, for the prediction and early diagnosis of diabetes mellitus (DIAPASON-DIAbetes Prediction And Screening Observational study). The DIAPASON protocol was approved by the institutional review boards of the IRCCS MultiMedica [protocol number 24/2012(153)]. Participants signed informed consent prior to laboratory screening. The thickness of the GCC was measured by OCT apparatus (Cirrus HD-OCT, Zeiss) analyzing an area of 6 × 6 mm of six scanned sectors around the fovea in both eyes. Exclusion criteria for the OCT analysis were visual defects (refraction ≥ 4 diopters) or concurrent glaucoma, keratoma, and macular degeneration.

### RNA Extraction and microRNA Expressional Analysis

For RNA extraction, 100 ul of plasma samples collected in ethylene di-amine tetra-acetic acid (EDTA) anticoagulant tubes were processed using miRNeasy Mini Kit (Qiagen, Hilden, Germany). AfterTaqMan microRNA reverse transcription, microRNa-15a expression was analyzed by the QuantStudio 6 Flex Real-Time PCR System (Applied Biosystems, Bradbury, NJ, USA) in total plasma and isolated EVs and normalized to the synthetic spike-in Cel-microRNA-39 (Qiagen, Hilden, Germany). The quantity of assayed microRNAs and Cel-microRNA-39 was retrieved relative to PCR data from a dilution curve of a known quantity input.

### EV Isolation and Quantification

One-hundred μL of plasma were used for the EV isolation with a ready-made chromatography method known to eliminate >95% of non-vesicular proteins (Exo-spin Blood, Cell Guidance Systems, Cambridge, UK) (22). EVs purity and quantity were measured by Nanoparticle Tracking Analysis, using Nanosight NS300 (Malvern Panalytical Ltd) (23).

### Statistical Methods

Categorical variables were compared using the χ^2^ test. Normally and not-normally distributed continuous variables were compared between diagnostic groups using the F-test or the non-parametric Kruskal–Wallis test. The linear regression model was used to evaluate the association between microRNA expression levels and diagnostic group (NGT, IGT, or T2DM) after adjustment by age and sex. The area under the receiver operating characteristic curve (AUC) was used to determine the discriminatory capability of microRNA-15a and HbA1c (mmol) with respect to the diagnosis of T2DM or IGT. AUCs were compared using the DeLong's test. The correlation between microRNA levels and clinical parameters was evaluated by the Spearman correlation coefficient. For each EV size, we estimated EV geometric mean concentrations in NGT, IGT and T2DM groups, adjusting for age and sex with multiple linear regression models. In addition, EV count for each size was naturally log-transformed to approximate normality of residuals. Due to the high number of comparisons, we used a multiple comparison method based on Benjamini–Hochberg False Discovery Rate (FDR) to calculate the FDR *P*-value. The association between OCT parameters and diagnostic groups and the association between microRNA levels and OCT parameters were evaluated on eye-specific data. All reported *p*-values were two-sided. A *p* < 0.05 was considered statistically significant. Statistical analyses were performed with SAS 9.4 statistical software (SAS Institute Inc., Cary, NC).

## Data Availability Statement

The datasets generated for this study are available on request to the corresponding author.

## Ethics Statement

The studies involving human participants were reviewed and approved by MultiMedica, Milan, Italy. The patients/participants provided their written informed consent to participate in this study.

## Author Contributions

ES contributed to the scientific hypothesis, handled the biological samples, and performed RT PCR analyses of microRNA-15a. ET performed the statistical analyses and drafted the manuscript. LS and FP participated to database construction, result interpretation, and paper writing. AU provided clinical data on the study population, collected and stored plasma samples, and populated the electronic database. PC actively participated to data discussion and interpretation and critically revised the manuscript. SL contributed to the article by supporting the collection of the clinical data and storing plasma samples. DS and FL contributed the article by collecting OCT clinicaldata, in addition to actively discussing, and interpreting results. LC acquired data of EV. CF contributed by performing statistical analysis of EV. PM retrieved funding, contributed in writing, and revising the manuscript. VB contributed by participating to result discussion and funding. SG contributed by generating ideas, participating in the scientific discussion on the structure and data of the paper, and participating in the drafting of the article. GS contributed to the article by establishing the hypothesis and research protocol of this study, drafting and revising the manuscript, and by retrieving funding.

## Conflict of Interest

The authors declare that the research was conducted in the absence of any commercial or financial relationships that could be construed as a potential conflict of interest.
